# New Modularity of DAP-Kinases: Alternative Splicing of the DRP-1 Gene Produces a ZIPk-Like Isoform

**DOI:** 10.1371/journal.pone.0017344

**Published:** 2011-03-08

**Authors:** Yishay Shoval, Hanna Berissi, Adi Kimchi, Shmuel Pietrokovski

**Affiliations:** Department of Molecular Biology, Weizmann Institute of Science, Rehovot, Israel; University of Poitiers, France

## Abstract

DRP-1 and ZIPk are two members of the Death Associated Protein Ser/Thr Kinase (DAP-kinase) family, which function in different settings of cell death including autophagy. DAP kinases are very similar in their catalytic domains but differ substantially in their extra-catalytic domains. This difference is crucial for the significantly different modes of regulation and function among DAP kinases. Here we report the identification of a novel alternatively spliced kinase isoform of the *DRP-1* gene, termed DRP-1β. The alternative splicing event replaces the whole extra catalytic domain of DRP-1 with a single coding exon that is closely related to the sequence of the extra catalytic domain of ZIPk. As a consequence, DRP-1β lacks the calmodulin regulatory domain of DRP-1, and instead contains a leucine zipper-like motif similar to the protein binding region of ZIPk. Several functional assays proved that this new isoform retained the biochemical and cellular properties that are common to DRP-1 and ZIPk, including myosin light chain phosphorylation, and activation of membrane blebbing and autophagy. In addition, DRP-1β also acquired binding to the ATF4 transcription factor, a feature characteristic of ZIPk but not DRP-1. Thus, a splicing event of the DRP-1 produces a ZIPk like isoform. DRP-1β is highly conserved in evolution, present in all known vertebrate *DRP-1* loci. We detected the corresponding mRNA and protein in embryonic mouse brains and in human embryonic stem cells thus confirming the *in vivo* utilization of this isoform. The discovery of module conservation within the DAPk family members illustrates a parsimonious way to increase the functional complexity within protein families. It also provides crucial data for modeling the expansion and evolution of DAP kinase proteins within vertebrates, suggesting that DRP-1 and ZIPk most likely evolved from their ancient ancestor gene DAPk by two gene duplication events that occurred close to the emergence of vertebrates.

## Introduction

**Figure 1:**
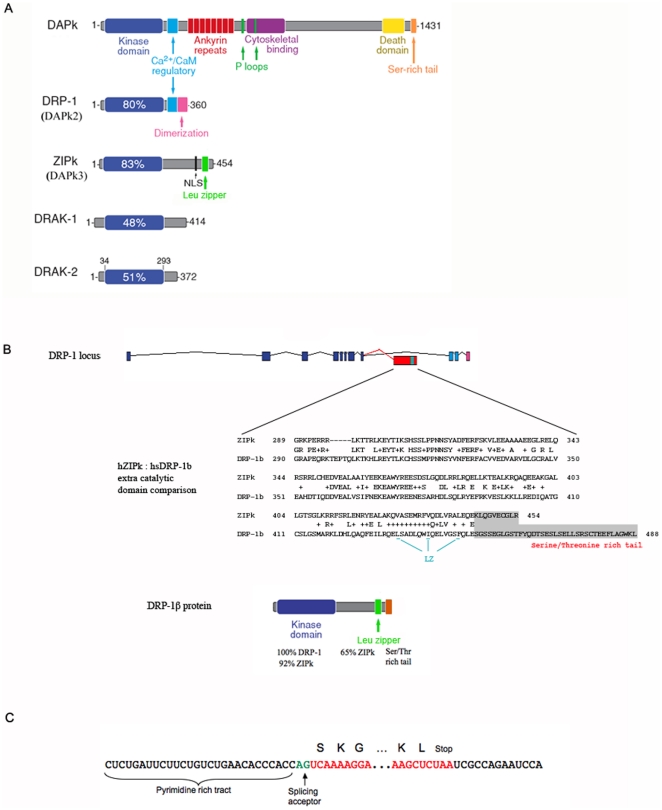
The DAPk family of proteins and the new member, DRP-1β. A. The percentage in the blue boxes, representing the catalytic domain of the kinases, indicates the extent of identity of each catalytic domain to the kinase domain of DAPk. B. A scheme of the genomic locus of DRP-1, DRP-1β exon and the DRP-1β protein structure. Dark blue- catalytic domain coding exons; light blue- CaM binding domain encoding exons, pink- dimerization tail encoding exons; red and green- the alternative open reading frame. Percents indicate similarity of the catalytic or extra-catalytic domain to the indicated protein. Enlarged area shows sequence alignment of the human alternative exon and the extra catalytic domain of human ZIPk. Letters indicate identities, pluses indicate similarities. Gray background indicates a non aligned area. LZ- leucine zipper. C. DNA sequence at the 5′ and 3′ of human DRP-1β alternative exon. Red- open reading frame; Green- splicing acceptor site. Capital letters- translated amino acids.

The Death Associated Protein Kinase (DAPK) family of proteins is a family of five Ser/Thr kinases which are very similar in their catalytic domain and are involved in programmed cell death (PCD) mechanisms. Three members including DAPk (also called DAPK1), DRP-1, (also called DAPK2), and ZIP-kinase (ZIPk, also called DAPK3), share about 80% identity in their catalytic domains thus creating a sub-family which is in the focus of this work. Two other members, DAPk Related Apoptosis inducing Kinase 1 and 2 (DRAK1 and DRAK2) are more distantly related, sharing only about 50% identity with DAPk [1]; also see Figure 1A).

DAPk is a 160 kDa, multi domain Ca^+2^/Calmodulin (CaM) regulated, Ser/Thr kinase. In addition to the catalytic and the CaM regulatory domains, it possesses several ankyrin repeats, a potential P loop motif, a cytoskeleton binding domain, a death domain and a C-terminal Serine rich region (Figure 1A). Ectopic expression of DAPk (as well as of ZIPk and DRP-1) induces membrane blebbing and cellular rounding through the phosphorylation of the regulatory light chain of myosin II (MLC). DAPk is activated by dephosphorylation of a specific site in the CaM regulatory domain and by Ca^+2^/CaM binding [2]. DAPk is involved in several pathways leading to cell death, including apoptosis, autophagy and anoikis-like cell death. It mediates several types of stress signals induced by IFN-γ TNF-α, Fas, TGF-β, ceramides, deprivation of neuronal cells from Netrin-1, and stimulation of NMDA receptors in cerebral ischemia [3], [4]. The gene is frequently silenced in cancer by promoter DNA methylation, suggesting that it functions as a tumor suppressor [5]. Moreover, a germline mutation in the human *DAPK1* promoter leads to a familial case of Chronic Lymphocytic Leukemia CLL [6]. DAPk may have also other functions, not related to PCD, such as a role in cytokinesis and cell migration [7], [8], [9], [10]. The *DAPK1* gene is well conserved in evolution from various invertebrates, such as C. elegans [11], to chordates and mammals. DRP-1 is a 42 kDa cell death-promoting kinase. Like DAPk, it contains a CaM regulatory domain which shares high sequence and functional similarity with that of DAPk, but its C-terminus differs completely from DAPk, possessing a unique 40 amino acid tail at its C terminus necessary for stabilizing the homo-dimerization state of the kinase [12]. Full activation of DRP-1 depends on relieving the inhibitory effects of the CaM regulatory domain by its binding to Ca^+2^/CaM and by the dephosphorylation of an critical Ser residue in this domain similar to DAPk regulation. In addition, homo-dimerzation is also necessary for full activation of the DRP-1, as long as the CaM regulatory domain is present [12]. DRP-1 is a cytoplasmic protein, and upon ectopic expression it induces autophagy, and caspase-independent autophagic cell death [8]. TNF-α induces both dephosphorylation and dimerization of DRP-1 and a functional interaction between DRP-1 and DAPk has been proposed as well [8], [12], [13], [14].

The third member of the DAPk family is ZIP kinase, a 55 kDa, Ser/Thr kinase. ZIPk-induced cell death can involve both caspase dependent and independent pathways, the former being mitochondrial dependent [15]. Unlike DAPk and DRP-1, ZIPk is not regulated by Ca^+2^/CaM. It contains a leucine zipper like domain at the C-terminus, needed for homo-oligomerization that is critical for its death promoting effects. ZIPk also contains a nuclear localization signals (NLS) and is localized both in the nucleus and the cytoplasm. Quite surprisingly it was recently found that the murine orthologs of ZIPk underwent a unique type of sequence divergence compared to other vertebrate species. As a consequence they are localized exclusively to the nucleus and also acquired several additional changes to compensate for their divergence [16]. A non-PCD function of ZIPk was observed in smooth muscle cells, where ZIPk-dependent phosphorylation of MLC led to Ca^+2^ sensitization and smooth muscle contraction. This was attributed to direct phosphorylation of MLC as well as inactivation of Smooth Muscle Myosin Phosphatase (SMMP-1M), through phosphorylation of the phosphatase's myosin binding subunit, and phosphorylation of its inhibitor protein CPI17 [17], [18]. Both DRP-1 and ZIPk genes are only present in vertebrates.

Previously it has been shown in our lab that there is a physical and functional cross talk between ZIPk and DAPk. DAPk is able to trans-phosphorylate ZIPk on six distinct sites in the extra-catalytic domain, thus increasing the cytoplasmic localization of ZIPk and the homo-trimerization towards a more potent cell death inducer. Accordingly, co-expression of both kinases causes a synergistic effect in promoting the membrae blebbing phenotype [15], [19], [20], [21], [22], [23]. These data, together with the epistatic relationship mentioned above, imply that the DAPk family may have a signaling capacity greater than the sum of signaling attributed to its individual members, perhaps even creating a cell death inducing kinase-kinase cascade.

In this work we illustrate an additional level of complexity in which transcripts derived from the genomic locus of *DRP-1* can undergo alternative splicing to give rise to a new kinase isoform, found to be expressed in embryonic stem cells and brain tissues. The alternatively spliced exon is homologous and highly similar to the C-terminus of ZIPk, thus generating a novel DRP-1 kinase isoform which shares functional characteristics with ZIPk. The DRP-1 gene organization, and its potential for alternate isoforms, is conserved in all known DRP-1 loci. Together with known sequences of other DAP kinases this provides an evolutionary model for the expansion and evolution of these kinases within vertebrates, and suggests that the DAP kinases sequence diversion is accompanied by retained sequence features.

## Results

### The genomic locus of *DRP-1* contains an alternative exon

Analyzing genomic loci of *DRP-1* (*DAPk2*) we identified a previously unknown putative exon. The exon is found in all vertebrate *DRP-1* gene loci that have been sequenced (Figure S1), is well conserved, and codes for 165–220 amino acids. This region is significantly similar to only one protein in the current sequence databases, to the extra catalytic domain of ZIPk (DAPK3), which is also encoded by a single exon. The new *DRP-1* exon is located between its previously known exons 8 and 9, immediately downstream of the catalytic domain encoding exons, and upstream of the two exons coding for DRP-1 regulatory extra catalytic domain (Figure 1B). All *DRP-1* loci, from fish to mammals, include a tightly conserved splice acceptor sequence at the 3′ end of the upstream intron (Figure 1C), and a stop codon in a well conserved position. Sequence analysis of the coding capacity of the exon shows its conservation pattern is typical to protein coding regions and includes numerous codons undergoing purifying selection (data not shown). Thus *DRP-1* has the potential to encode another isoform to its previously known product, where an alternative splicing event will replace the known CaM regulatory and dimerization domains with a ZIPk-like extra catalytic domain.

**Figure 2:**
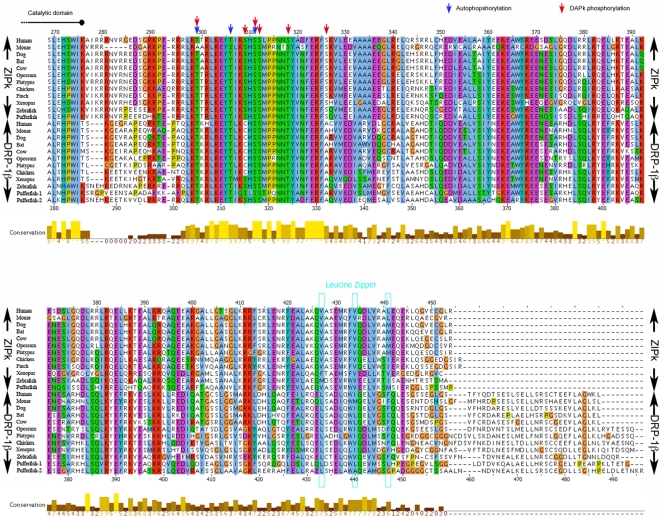
The DRP-1β alternative exon shows similarity to the extra-catalytic domain of ZIPk. A multiple sequence alignments of the extra catalytic domain of ZIPk and DRP-1β orthologs from the indicated vertebrates. Blue arrows- ZIPk autophosphorylation sites; red arrows- ZIPk phosphorylation sites by DAPk. Brown to yellow bars- conservation measure; the position of ZIPk leucine zipper is marked by pale blue boxes. The MSA was performed using CLUSTALW program and visualized with the JalView tool.

The human *DRP-1* exon we identified is 202 amino acids long. Residues 5–165 of this exon have 42% identity (and 65% similarity) to residues 289–444 of human ZIPk (Figs. 1B and 2). The C-terminal 37 amino acids of the exon are Ser/Thr rich, with no significant similarity to any known protein in the current sequence databases. Thus, in case of an alternative splicing event at the locus of *DRP-1*, the predicted translated protein would be very similar to ZIPk- e.g., in humans 79% identity in the catalytic domain and 42% identity in the extra catalytic domain (Fig. 1B). Unlike DRP-1, this protein is not expected to be regulated by calcium, as it lacks its Ca^+2^/CaM binding domain. Detailed analysis of available sequence data identified transcripts of the new isoform in pig, cow, chicken and several fish (Table S1). We termed the alternative spliced isoform DRP-1β.

### Sequence analysis of DRP-1β alternative exon

To further study the features of the DRP-1 new exon, we aligned all the protein sequences we found for it and compared the alignment to a similar alignment of the ZIPk extra catalytic domain (Figure 2). The two regions are very similar and can be confidently aligned across their N-terminal 80%. The most conserved region is at human ZIPk positions 297–332. ZIPk contains at this region several sites that are phosphorylated by DAPk and several autophosphorylation sites, shown to be important for full activation of the protein [21], [24]. Most of these sites are conserved in DRP-1β (Figure 2) and may undergo similar regulation. Another conserved region corresponds to the Leucine zipper-like motif of ZIPk, at position 433–447 of DRP-1β, especially due to the presence of hydrophobic amino acids at the key positions 433/440/447 of the heptameric repeat that creates the zipper itself [25] (Figure S2). Sequence prediction for coiled coil domains showed both these DRP-1β and ZIPk regions to most probably adopt this structure, as expected for Leucine Zipper type dimerization regions (data not shown).

It is interesting to note that unlike ZIPk, DRP-1β is conserved in murines, and did not undergo the murine-specific divergence characteristic of murine ZIPk which we have previously described [16]. Thus, while the extra catalytic domain of mouse ZIPk shows only 81% similarity to that of human ZIPk, mouse alternative exon DRP-1β is 92% similar to its human ortholog, This suggests that DRP-1β has a distinct, separate role from ZIPk, and thus was not under the same evolutionary pressure which led ZIPk to diverge from the common consensus in murines.

### mRNA and protein expression of DRP-1β

**Figure 3:**
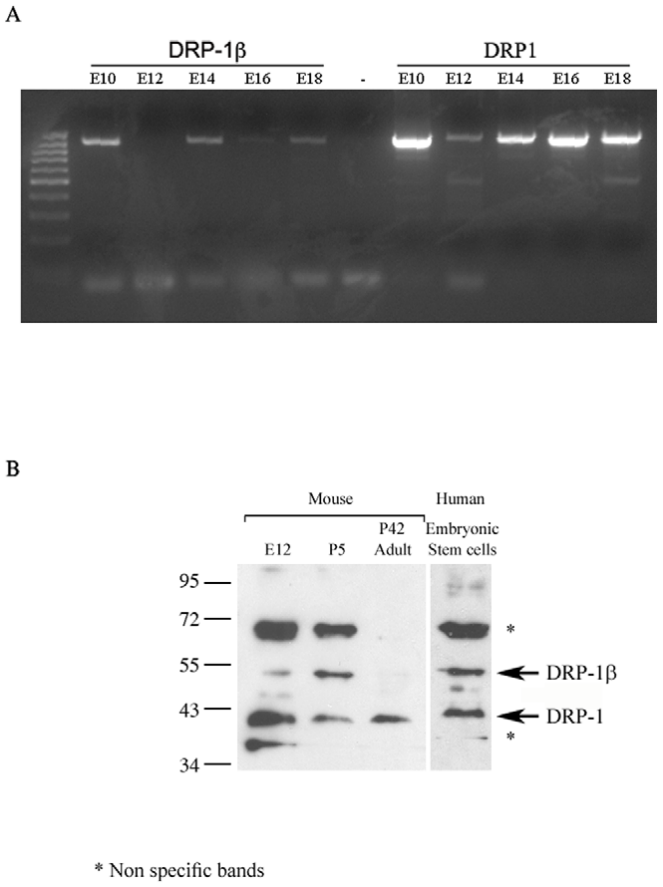
mRNA and protein expression of DRP-1β. A. DRP-1β and DRP-1 mRNA fragments were amplified by PCR, using total embryo mouse cDNA from the indicated days as template, followed by ethidium bromide gel detection. B. Western blot detection of DRP-1β and DRP-1 protein levels in brain tissues of mice and human embryonic stem cells, using anti N'-DRP-1 antibody. E12- embryonic day 12, P5 - postnatal day 5, P42 – postnatal day 42 (adult mouse).

Database searches identified a few DRP-1β expressed sequence tags (ESTs) suggesting that this isoform may be expressed in some settings (Table S1). Yet the low number of DRP-1β ESTs stands in contrast to the numerous ESTs of DAPk, DRP-1 and ZIPk reported to date (Table S1), suggesting that unlike the ubiquitous expression of the three well known family members the new alternate isoform may have a more restricted pattern of expression. Experimentally, we identified the presence of DRP-1β mRNA in cDNA libraries of mouse embryos. The detection was done by PCR amplification, using specific probes on both sides of the alternative splice junction. DRP-1β mRNA was clearly detected in samples from embryonic days 10, 14, 16 and 18 (Figure 3A), proving the presence of this alternatively spliced mRNA in embryonic cells of different developmental stages (Note that the apparent lack of detection of DRP-1β mRNA in samples from day 12 is due to the fact that the quality of the sample is lower, as shown by the attenuated detection of DRP-1 mRNA, used as a control (Figure 3A)). We next searched for DRP-1β protein expression, using antibodies directed against the N' terminus of DRP-1 (which is present in both isoforms), and which recognize both human and mouse proteins. The isoform distinction is done through the size of the protein, where DRP-1 runs on gels as a 42 kD band [13] and DRP-1β is predicted to display a size of 55 kD. Initial screen of various human and mouse cells lines, including HEK293T, HeLa, H1299, and NIH3T3, failed to detect a band of the appropriate size. We next screened brain extracts from fetal and young mice, and found a strong signal at the expected size, that was absent in adult mouse brains, suggesting strong protein expression of the DRP-1β isoform in the brains of embryos and young mice (Figure 3B). DRP-1 on the other hand was expressed in all the brain samples taken. DRP-1β isoform was also detected in human embryonic stem cells (Figure 3B). Thus, we proved that the *DRP-1* locus undergoes alternative splicing in some tissues/cells from early developmental stages, and that the alternative transcript is translated into protein, both in mice and humans.

### Functional characterization of DRP-1β protein

**Figure 4:**
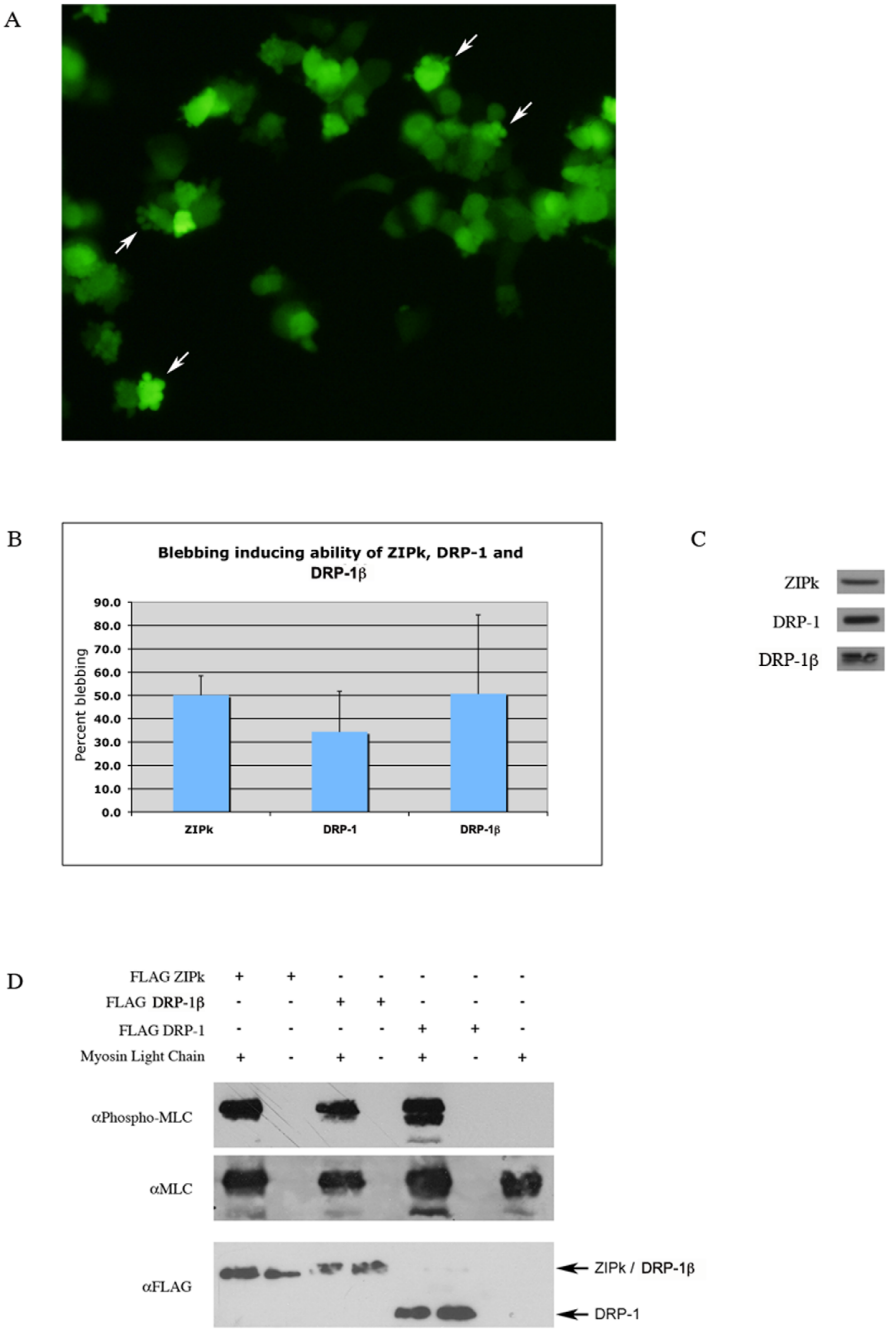
Ectopically expressed DRP-1β induces MLC phosphorylation and membrane blebbing in cells. A. DRP-1β ectopic expression induces membrane blebbing. HEK293T cells were co-transfected with FLAG DRP-1β and GFP expression vectors, and examined under fluorescent microscope after 24 h. White arrows- cells exhibiting membrane blebs. B. Quantification of the blebbing inducing ability of ZIPk, DRP-1 and DRP-1β. Note that the number of blebbed cells in cells transfested with control plasmids is below detection levels C. Western blot detection of the kinases, (detection was done with anti-FLAG Abs, running the samples in the same gels and the same exposure time of the blots) indicating comparable expression levels. D. ZIPk, DRP-1β and DRP-1 phosphorylate myosin light chain (MLC). FLAG tagged kinases were expressed in HEK293T, immunoprecipitated using anti-FLAG antibodies and eluted from beads. His-tagged MLC was purified from bacteria, and used as a substrate in an in vitro kinase assay. MLC phosphorylation was detected using an antibody against phospho-serine 19 on MLC.

To characterize the properties of the DRP-1β protein we cloned FLAG-tagged DRP-1β in a mammalian expression vector. Over-expression of the protein in 293T cells led to extensive membrane blebbing (Figure 4A), at levels comparable to those induced by over-expression of DRP-1 and ZIPk (Figure 4, B and C).

DAP kinase family of proteins induce membrane blebbing by phosphorylating the regulatory light chain of myosin II, MLC [1]. To verify that DRP-1β retains this ability, we performed an in vitro kinase assay, using MLC as substrate. As shown in Figure 4D, DRP-1β can phosphorylate MLC on serine 19 to a level comparable to ZIPk and DRP-1.

**Figure 5:**
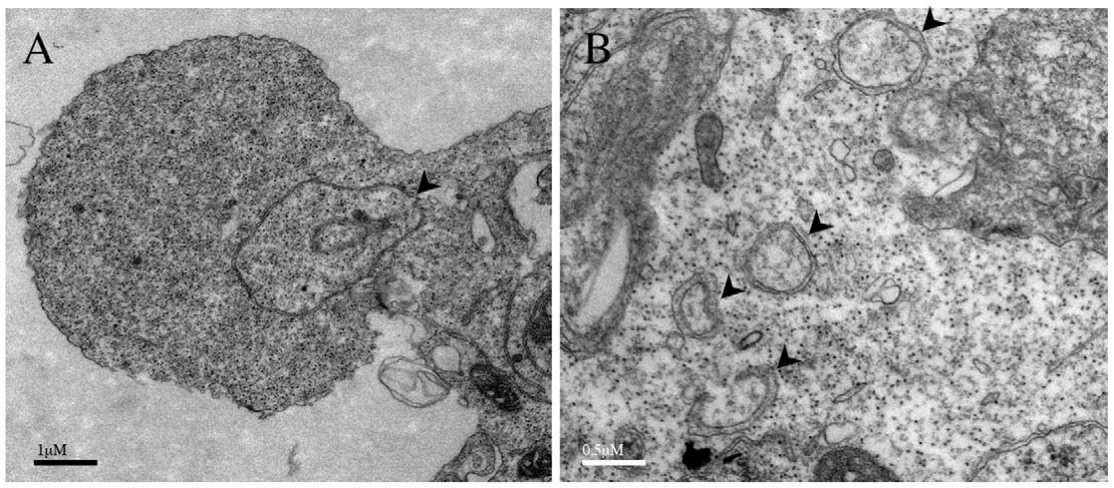
Ectopic expression of DRP-1β induces the accumulation of autophagic vesicles. HEK293T cells were transfected with DRP-1β expression vector, fixed 24 h after transfection and examined using Transmission Electron Microscopy (TEM). A. a cell undergoing membrane blebbing; B. larger magnification of induced vesicles. Arrowheads indicate double membrane, autophagic vesicles.

Transmission electron microscopy (TEM) studies were next performed to find out whether DRP-1β induces the accumulation of autophagosomes like the other members of the DAPk family. It was found that double membrane vesicles characteristic of autophagosomes were clearly evident upon DRP-1β transfection; the autophagosomes were detected at high number within the membrane blebs (Figure 5A) and the cell body (Figure 5B). This is consistent with the autophagic phenotype induced by DRP-1 and ZIPk ([8], [21], and Kimchi et al., unpublished data). Western blot analysis revealed that the ectopic expression of each of the three kinases induced the conversion of LC3-I to the lipidated LC3-II form which is a marker of autophagy activation (Figure S3) while none of them activated caspases, a marker of apoptosis (data not shown).

Altogether, the ectopic expression experiments indicate that DRP-1β shares some biochemical and cellular properties with both DRP-1 and ZIPk, including MLC phosphorylation, membrane blebbing and autophagy.

### DRP-1β and ZIPk but not DRP-1 share a common interacting protein

Since DRP-1β shows high similarity to ZIPk, we next examined whether it retains some of the ZIPk unique characteristics. The high degree of conservation of the leucine zipper-like motif of ZIPk in DRP-1β led us to examine whether both proteins can interact with the same partners through this structural domain. Activating transcription factor 4 (ATF4) was previously shown to bind ZIPk through its leucine zipper [25] and was thus selected for this study.

**Figure 6:**
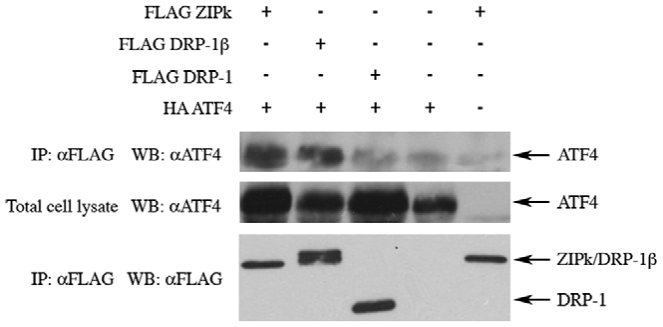
ZIPk and DRP-1β bind ATF-4, while DRP-1 fails to do so. HEK293T cells were co-transfected with the indicated vectors and harvested 24 h post transfection. Lysates were immunoprecipitated using anti-FLAG antibodies, and protein levels were detected using western blot.

To this end, we performed a co-immunoprecipitation experiment to examine whether the ectopically expressed DRP-1β and ATF4 proteins interact with each other. As shown in Figure 6, both ZIPk and DRP-1β were able to pull down ATF4, while DRP-1 could not. Thus, DRP-1β shares at least one interacting protein with ZIPk, a function gained by the alternative splicing which does not exist in the canonical DRP-1 isoform. A leucine zipper mutant of DRP-1β, in which three key hydrophobic amino acids (at the d position of the heptamer in Figure S2) were substituted to alanines, displayed a reduced ability to pull down ATF4 ().

### DRP-1 and ZIPk evolved from DAPk at the emergence of jawed vertebrates

**Figure 7:**
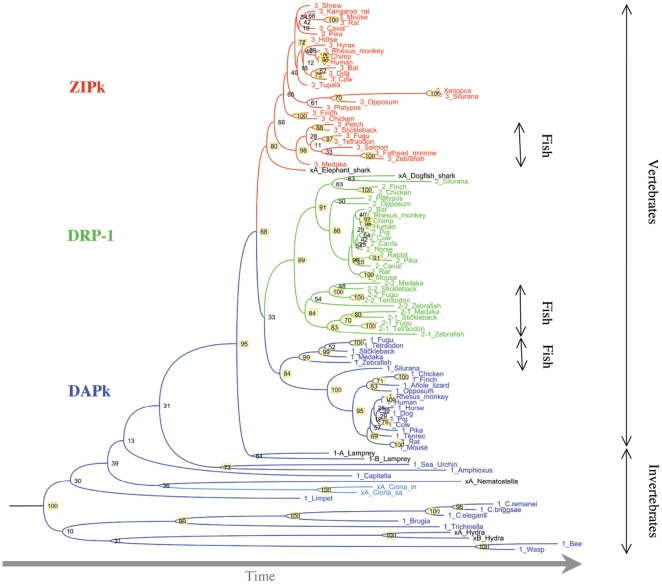
The DAP kinases phylogenetic tree. A phylogenetic tree of the indicated organisms was constructed based on the multiple alignment of the DAP kinase scatalytic domain, using the PHYML program. Numbers above branches represent bootstrap support from 100 replicates. Yellow background- high bootstraps value. Blue- DAPk; Green- DRP-1; Red- ZIPk; Black- ortholog undetermined due to partial sequence.

To find out the relation between the different DAPK family members we calculated a phylogenetic dendogram from an alignment of the kinase catalytic domains from DAPk, DRP-1 and ZIPk. To determine the root of the dendogram and its time dimension we included DRAK proteins that are more distant from DAPk, DRP-1 and ZIPk proteins than the distance in between these members [1], [26]. (Figure 7)

The resulting dendogram clearly separates the DAPk, DRP-1 and ZIPk proteins from each other, and within each cluster most sequences are grouped according to accepted taxonomic relations of the species they are found in. DAPk proteins are most diverse, confirming their identification in both invertebrates and vertebrates. DRP-1 and ZIPk clusters appear on the dendogram next to the vertebrate DAPk cluster. The closest cluster to these three vertebrate clusters is a clearly separated (bootstrap value of 95/100) and earlier branching cluster with two lamprey sequences. The sequences from this jawless vertebrate have a DAPk kinase domain (but their extra catalytic domain is yet undetermined), and their dendogram position is in between the jawed vertebrate DAPk proteins and the DAPk proteins of other invertebrates, simpler chordates (i.e, Amphioxus), and urochordates (i.e, Ciona). DRP-1 and ZIPk clusters each include a sequence from a shark species. The Elephant shark (*Callorhinchus milii*) includes at least five *DAPk* genes but their publicly available sequences are partial, highly fragmented, and most are too short to include in phylogenetic dendograms (SP, data not shown).

Ciona species belong to a basal urochordate sub phyla that diverged before the emergence of vertebrates [27]. Two Ciona species *C.intestinalis* and *C.savignyi* include a DAPk protein with an extra catalytic domain different from those of DAPk, DRP-1 and ZIPk. These proteins are clearly placed within the DAPk cluster on the dendogram, showing them to be a novel “offshoot” of these proteins. Other interesting DAPk sequences appear in the insects Honeybee (*Apis mellifera*), Jewel wasp (*Nasonia vitripennis*), ants (*Camponotus floridanus*, *Harpegnathos saltator*, *Atta cephalotes*), and Red flour beetle (*Tribolium castaneum*). No DAPk sequences were identified in any of the Drosophila and mosquito genomes sequenced so far, but probable DAPk sequences are found in other arthropods including insects, arachnids, and crustaceans (Table S1). It thus seems most likely that the *DAPk* gene was lost in a dipteran progenitor of mosquitos and flies. Partial sequences of DAPK-like kinase domains from cnidaria, basal metazoa with radial symmetry, could be found and cluster with the invertebrate DAPk sequences. However, they are not all clustered together and their exact nature would be better understood once their full sequences will be available.

### Fish *DRP-1* genes

**Figure 8:**
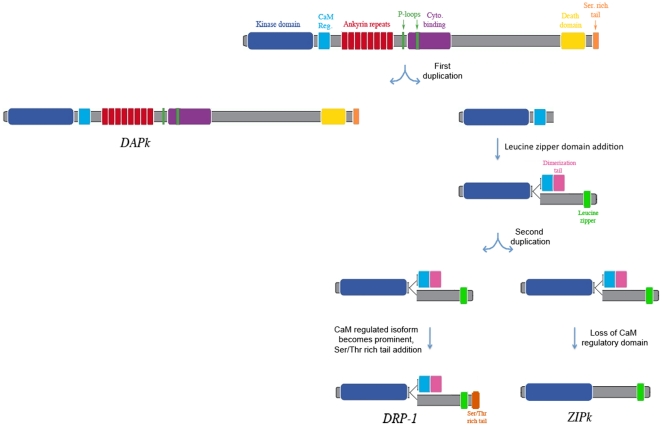
A model of the Evolution of the DAP kinases. Scheme showing a most-parsimonious model of the evolution of DRP-1 and ZIPk in vertebrates.

Fish exhibit a unique composition of DAP kinases. Two *DRP-1* gene loci were found by us in each of six different teleost fish species where relatively large genomic and EST sequence data is available. These two *DRP-1* sub-type groups cluster together (Figure 8) and are the probable result of the whole genome duplication that occurred after the divergence of ray-finned fish [28]. One fish *DRP-1* sub type has the same genomic structure as the *DRP-1* gene in other vertebrates, i.e., including 3′ exons coding for both the previously known DRP-1 and ZIPk-like extra-catalytic domains. The second fish *DRP-1* sub type gene only has the exon for the ZIPk-like domain, missing the previously known *DRP-1* 3′ exons (Figure 1A). In zebrafish there is another change - its first *DRP-1* sub type gene does not have a C-terminal ZIPk-like exon. Thus, the two zebrafish *DRP-1* genes each have a different extra-catalytic domain.

It is possible that in the teleost fish, *DRP-1* gene has evolved, or is even yet evolving, into two distinct genes. The function, or at least coding capacity, of these duplicated *DRP-1* genes could be equivalent to the different transcripts of the single *DRP-1* gene of other vertebrates. It is also interesting that we found no evidence in fish for *DAPk* and *ZIPk* gene duplicates. Perhaps the two *DRP-1* gene duplicates were retained after the fish whole genome duplication because of the distinct alternative messages possible from their progenitor gene.

## Discussion

Here we report the discovery of a novel member of the DAP kinase family DRP-1β, highly conserved from fish to mammals, which is generated by alternative splicing event of the *DRP-1* gene. The uniqueness of this isoform is in its close resemblance to ZIPk, another member of the DAP kinases family, due to an interesting modular organization discovered here. The resulting modular cross similarity within the DAP kinase proteins could allow for intricate control of their function and is unique, to the best of our knowledge.

We show that this alternative splicing takes place in mouse embryonic tissues and that the product of the new isoform is expressed in the embryonic brains of mice and human embryonic stem cells. We further show that DRP-1β is an active kinase, able to phosphorylate MLC on serine 19 and induce membrane blebbing, and autophagic vesicle formation. Further studies will need to be conducted in order to determine the physiological roles of DRP-1β and the specific differences between this kinase and the other DAP kinases, that appear to have kept the relative complicated DAP kinase genes arrangement throughout vertebrate evolution. It is interesting to note that a recent paper by Tang et al. shows that ZIPk plays a role in induction of autophagy by phosphorylating the ULK1 protein [29]. It should be examined if DRP-1β can perform the same function.

The interaction of DRP-1β and ATF-4 has unclear functional implications, as the function of the interaction of ZIPk and ATF-4 is not known either. It is possible that these interactions sequester ATF-4 from the nucleus and its genomic targets, thus halting the induction of pro-survival genes. On the other hand, it has been suggested that ATF-4 may block the pro-death activities of ZIPk by preventing its homo dimerization [25], and it may play a similar role for DRP-1β. These possibilities should be further pursued in order to establish them. In any case, this interaction proves that DRP-1β, although possessing the DRP-1 kinase domain, has functions similar to those of ZIPk. DRP-1β has also lost some of the DRP-1 features, like the regulation by Ca^+2^/CaM. The conservation of DRP-1β and ZIPk and their gene organization across vertebrates, perhaps even from the very emergence of this subphylum, excludes complete redundancy and indicates some essential and specific roles for both kinases. On the one hand the localization of the ZIPk like extra-catalytic exon in the same genetic locus of DRP-1 may allow a coordinated control of both DRP-1 and DRP-1β as opposed to their placement in separate genomic loci. On the other hand, the additional presence of *ZIPk* on a distinct locus is assumed to have a significant advantage suggesting some unidentified yet functional difference between ZIPk and DRP-1β. The two genes are driven by different promoters, and their extra-catalytic domains are not completely identical (Figure 2).

The discovery of a ZIPk-like encoding exon sheds light on the evolution of the entire DAPK family, and enables us to hypothesize on the events leading to the emergence of *DRP-1* and *ZIPk* genes. One parsimonious course of evolutionary events that could have led to the present nature and distribution of DAPk genes is shown in Figure 8. DAP-kinase emerged early in the evolution of metazoa, appearing already in nematodes, flat worms and other bilateral invertebrates as a single *DAPk* gene in their genomes. Around the development of the jawed vertebrates, the *DAPk* gene underwent a duplication with one copy losing the extra catalytic coding regions downstream of the CaM regulatory domain and acquiring (from an unknown source) two single coding-exon regions for protein dimerization. A region coding for a leucine-zipper like dimerization domain was inserted between the catalytic domain exons and the CaM regulatory domain, and a much shorter exon coding for a different dimerization domain was inserted downstream of the CaM regulatory domain. We have no data at present to determine if this happened in a single or multiple events. The resulting gene progenitor of *DRP-1* and *ZIPk* then underwent a second gene duplication. One gene lost the Ca^+2^/CaM and adjacent dimerization exons, creating the *ZIPk* progenitor. The second gene kept all exons, but probably expressed either the leucine zipper encoding exon or the 3′ Ca^+2^/CaM and dimerization exons by alternate splicing, thus giving rise to DRP-1 and DRP-1β isoforms.

In conclusion, our integrated research approach and analysis of diverse data allowed us to identify a complex case of gene evolution and expression. Examining all available *DRP-1* loci confirmed the coding nature of the cryptic exon which we found. This, together with subsequent experimental data, transformed, in turn, the absence of human and mouse ESTs for this exon, from a trivial and uninformative observation to a hypothesis for a restricted and potentially interesting expression of a new DRP-1 isoform. Identifying the DRP-1β isoform in the brain of embryonic and young mice excluding the adult phase may have functional implications for our future understanding of why such domain modularity evolved in this family of death –inducing kinases.

## Materials and Methods

### Sequences

Sequences accession numbers and compositions are detailed in the supplementary material.

### Multiple sequence alignments and Phylogenetic tree

Multiple sequence alignments were generated using the BLAST [30], DIALIGN2 [31], GLAM2 [32], and LAMA [33] programs. Sequence reads were assembled using the CAP3 program [34]. Sequences were aligned and edited analysis with Se-Al (http://tree.bio.ed.ac.uk/software/seal/) program. Sequence dendograms were calculated based on the multiple alignment of the catalytic domains of DAPk, DRP-1 and ZIPk, and rooted using the catalytic domain of DRAK, using the PHYML v.2.4.4 program [35].

### Plasmids

Human DRP-1 and ZIPk plasmid were previously described [13], [21]. Human DRP-1β exon was amplified through PCR using genomic DNA as template, ligated to DRP-1 catalytic domain coding sequence and finally subcloned into a FLAG-tagged pcDNA3 expression plasmid. HA tagged human ATF4 expression plasmids were kindly provided by Prof. Michael S. Kilberg and by Prof. Fung-Fang Wang. The leucine zipper perturbation of DRP-1β was created by introducing the substitutions L433A/I440A/F447A to human DRP-1β, using the PCR site-directed mutagenesis protocol.

### mRNA and protein detection

DRP-1 and DRP-1β mRNA detection was performed using PCR amplification with cross-exon primers. Mouse embryo cDNA (MD-104, Zygen) of the indicated days was used as template. Anti-DRP-1 (N' terminus) Rabbit monoclonal antibody (dilution 1∶500) (AbCaM, EP1633Y) was used for endogenous DRP-1β protein detection in lysates of the indicated cells or tissues. Mouse brain tissues were kindly provided by Prof. Orly Reiner.

### Cell culture and transient transfection

293T Human Embryonic Kidney (HEK) cells and HeLa cells were grown in DMEM (Biological Industries) supplemented with 10% fetal bovine serum (Hyclone) and 1% L-Glutamine (GibcoBRL) and a mixture of antibiotics (100 u/ml penicillin and 0.1 mg/ml streptomycin). For transient transfections, 1.2×10^6^ (293T) or 0.8×10^6^ (HeLa) cells were plated on 9 cm plates 24 hours prior to transfection. Transfections were done by the calcium phosphate method with 10 µg DNA per plate. To assess the membrane blebbing potency of DRP-1β, DRP-1 and ZIPk, 293T cells were transfected with the appropriate plasmid and 1 µg of peGFP expression vector. After 24 hours, green cells were counted and the percent of blebbing cells was calculated. Western blot analysis was conducted to ensure equal kinase protein expression.

### Immunoprecipitation

Cells were washed twice in PBS and then suspended and vortexed in cold PLB lysis buffer (5 mM EDTA, 10 mM NaPO_4_, 1% Triton X-100, 0.1 M NaCl, 0.5% DOC, 0.1% SDS) with protease inhibitors (1% protease inhibitor cocktail (Sigma), 1% PMSF). Lysates were centrifuged for 15 min. at 14,000 rpm at 4°C. The pellet was discarded and the supernatant was pre-cleared for 1 hour at 4°C on a slurry of protein G-PLUS Agarose beads (Santa Cruz Biotechnology). The pre-cleared extracts were incubated with Agarose-conjugated anti-FLAG M2 gel beads (Sigma) for 2 hours at 4°C. Immunoprecipitates were washed 4 times with lysis buffer containing protease inhibitors, and resolved by standard SDS-PAGE. Blots were reacted with anti-FLAG-M2 monoclonal antibody (dilution 1∶500) (Sigma); anti-CREB-2 (ATF4) rabbit polyclonal antibody (dilution 1∶500) (Santa Cruz); or anti-Actin monoclonal antibody (dilution 1∶5000) (Sigma).

### *In vitro* kinase assay

Immunoprecipitated FLAG- DRP-1β, DRP-1 or ZIPk were quantified after elution against standards. 200 nmol bacterial expressed, purified, human MLC was incubated with or without 50 nmol kinase in reaction buffer (50 mM HEPES, pH 7.5, 20 mM MgCl_2_) containing 1 mM bovine calmodulin, 0.5 mM CaCl_2_ and 50 mM ATP. The kinase assay was conducted at 30°C for 30 minutes. Protein sample buffer was added to terminate the reaction, and after boiling, the proteins were analyzed resolved by standard SDS-PAGE. Kinase protein levels were detected using anti-FLAG-M2 monoclonal antibody (dilution 1∶500) (Sigma); Substrate protein levels were detected using polyclonal anti-MLC antibody (dilution 1∶300) (E201); MLC-phosphorylation was detected using polyclonal anti-phospho Ser19 MLC antibody (dilution 1∶1,000) (Cell Signaling).

### Immunostaining

0.8×10^6^ HeLa cells were seeded on glass cover slips in 9 cm plates and transfected the next day with the appropriate constructs, 10 µg DNA per plate. After 24 hours, cells were fixed in 3.7% formaldehyde for 15 min. After blocking and permeabilization with 10% normal goat serum (Biological Industries), 0.4% Triton X-100 in PBS, the cells were incubated for 1 h with anti-FLAG polyclonal antibody (Sigma; 1∶600 dilution) followed by RRX-conjugated goat anti-rabbit secondary antibody (Jackson ImmunoResearch; dilution 1∶800). The cover slips were finally stained with DAPI (0.5 mg/ml, Sigma) and mounted with ImmuMount (Thermo Shandon) embedding media. Stained cells were viewed by fluorescent microscopy (Olympus BX41) equipped with a 100x oil immersion objective, using excitation wavelengths of 530-550l (for RRX) and 360-370l (for DAPI). Digital imaging was performed with a DP50 CCD camera using Viewfinder Lite and Studio Lite software (Olympus). Final composites were prepared in Adobe Photoshop (Adobe Systems).

### Transmission Electron Microscope

293T cells were transfected with DRP-1β, DRP-1 or ZIPk expression plasmids. Cells were fixed for 1 hr in Karnovsky's fixative (3% paraformaldehyde, 2% glutaraldehyde, 5 mM CaCl_2_ in 0.1 M cacodylate buffer [pH 7.4], containing 0.1 M sucrose). Cells were scraped, pelleted, and embedded with agar noble to a final concentration of 1.7% and postfixed with 1% OsO_4_, 0.5% potassium dichromate, and 0.5% potassium hexacyanoferrate in 0.1 M cacodylate buffer. The pellet was stained en bloc with 2% aqueous uranyl acetate followed by ethanol dehydration and embedded in EMbed (EMS). Sections (75 nm) were cut, stained with 2% uranyl acetate in 50% ethanol and lead citrate, and examined using a T12 BioTwin (FEI Holand) transmission electron microscope at an accelerating voltage of 120 KV. Digital images were obtained with Eagle CCD 2K by 2K camera (FEI Holand).

## Supporting Information

Figure S1 **DRP-1β proteins alternatively-spliced extra catalytic region.** Most fish species included have two DRP-1β isoforms marked by 1 and 2. See text for discussion of the fish DRP-1β isoforms.
(DOCX)

Figure S2 **Conservation of the leucine zipper-like motif in DRP-1β and ZIPk.** Logo showing the conservation of the leucine zipper-like motif of both proteins. Upper case letters colors indicate amino acid sub group; lower case letters indicate the amino acid position in the α-helix structure, with the d position of the hydrophobic amino acid marked in red. Sequence logos were calculated according to reference [Henikoff, S., Henikoff, J. G., Alford, W. J., and Pietrokovski, S. (1995) Gene (Amst.) 163, GC17–GC26].
(TIF)

Figure S3 **Ectopic expression of DRP-1, ZIPk and DRP-1β induces LC3 shift.** HEK293T cells were transfected with the FLAG-tagged ZIPk, DRP-1β or DRP-1 expression vectors or were mock transfected with a nonrelevant protein expressing plasmind, and were harvested 24 h post transfection. Lysates were immunoblotted using anti-LC3, anti-FLAG and anti-Actin antibodies.
(TIF)

Figure S4 **The binding of DRP-1β to ATF-4 is through the leucine zipper-like domain.** HEK293T cells were co-transfected with the indicated vectors and harvested 24 h post transfection. Lysates were immunoprecipitated using anti-FLAG antibodies, and protein levels were detected using western blot with the indicated antibodies.
(TIF)

Table S1 **Sequences used in the multiple sequence alignment of the DAP kinases.** The table details organisms, sequence accession numbers and composition assembly of the sequences.
(DOC)
