# Supplementary material for: New Modularity of DAP-Kinases: Alternative Splicing of the DRP-1 Gene Produces a ZIPk-Like Isoform
Source: PLoS One. 2011 Mar 8;6(2):e17344. doi: 10.1371/journal.pone.0017344 (PMC3050894; doi:10.1371/journal.pone.0017344)
Supplement: Figure S1 — DRP-1β proteins alternatively-spliced extra catalytic region. Most fish species included have two DRP-1β isoforms marked by 1 and 2. See text for discussion of the fish DRP-1β isoforms. (DOCX) [file pone.0017344.s001.docx]

DRP-1β alternatively-spliced extra catalytic sequences

>human

SKGEGRAPEQRKTEPTQLKTKHLREYTLKCHSSMPPNNSYVNFERFACVV

EDVARVDLGCRALVEAHDTIQDDVEALVSIFNEKEAWYREENESARHDLS

QLRYEFRKVESLKKLLREDIQATGCSLGSMARKLDHLQAQFEILRQELSA

DLQWIQELVGSFQLESGSSEGLGSTFYQDTSESLSELLSRSCTEEFLAGW

KL*

>chimpanzee

SKGEGRAPEQRKTEPTQLKTKHLREYTLKCHSSMPPNNSYVNFERFACVV

EDVARVDLGCRALVEAHDTIQDDVEALVSIFNEKEAWYREENESARHDLS

QLRYEFRKVESLKKLLREDIQATGCSLGSMARKLDHLQAQFEILRQELSA

DLQWIQELVGSFQLESGSSEGLGSAFYQDTSESLSELLSRSCTEEVLAGW

KL*

>rhesus monkey

SKGEVRAPEERKTEPTQLKTKRLREYTLKCHSSMPRNNSYVNFERFACVV

EDVARVDLGFHALVEANDTIQDDVEALVSIFNEKEAWYREENESTRHDLS

QLRYEFRKVESLKKLLREDIQATGSSLGSVARKLDHLQTQFEILRQEFSA

DLQWIQELVGSFQLESGSSEGLGSVFHQDTSEPLSELLSRSCTEEVLAGW

KL*

>mouse

SKGEARAPEQWKAQPAQLKTKRLREYTLKCHSSMPPNNTYVNFERFAHVV

EDVARVDKGCRALAGAHDTLQDDVESLVSIYNEKEAWYREENENARHNLS

QLKYEYRKVESLKKLLREDIQATGASLGGVARKLDHLQAQFETLRQQLSA

DIQWMQELVGIFQLESENTDSHSLGFMFHRDPSESLSELLNRSHAEEVLA

GLSL*

>rat

SKGEASVPEQWKTQPAQLKTKRLREYTLKCHSSMPPNNTYVNFERFAHVV

EDVARVDEGCHALAGAHDTLQDDVESLVSIYNEKEAWYREENENARHNLS

QLKYEFRKVESLKKLLREDIQAAGASLGGVARKLDHLQVQFETLRQQLSA

DIQWMQEMAGILQLESGNTDGHALGSVFHRDTSESLPELLNRSHTEGILA

GLNL*

>American pica

LGGEASTPEQRKTQPPQLKTKHLREYTLKCHSSMPPNNTYVNFERFAHVV

EDVARADQECHALAGTHATLQDDVEALISIYNEKEAWYREENERARHDLS

QLRYELRKVQSLKKLLREDLRATSCSLGGVARKLDHVQEQFEALRRELSA

DLQWMQEVVSSFQPESGCTDKLGSMLRGGAAGTFSQLLSKACGDQVLGGV

QL*

>cavia

SKDETRAPEQRKTQTSQLKTKRLREYTLKCHSSMPPNNTYVNFEHFAQMV

EDIARVDQGCHALAGTHDTLQDDVETLISIYNEKEAWYREENESARHNLS

QLKYEFRKVKSLKKLLREDIRATGSSLGRVARKLDHLQAQFEALRQELSA

DLQWVQELASSFQLESRNADGLGSVLPWDTSESLSELPSGSHTEEVLASL

KL*

>rabbit

SGGEASAPEQRETEPPQLKTKRLREYTLKCHSSMPPNNTYVNFERFARVV

EDMARVDEGCRALAGAHDTLQDDVETLISIYNEKEAWYREENESARHDLS

QLRYEFRKVESLKKLLREDLQATSASLGGVARKLERVQEQFGALRRELSA

DLQWMQEVVNGFQLEGGRTDGLGSVFHGDASESLSELLSRVCSEEVLGTV

QL*

>cow

SKGEIRAPEQHKAEPNQLKTKRLREYTLKCHSSMPPNNTYINFERFARVV

EDVARVEQGCRALTGAHDTIQDDVETLISIYNEKEAWYREESERARHDLS

QLRYEFRKVKSSKKLLREDIRATGSRLGGAARKLDHLQTQFETLRQELSA

DLQWLQELVGSFQLESGSMDSPGSVFCRDASESFGELLNRSCGEEVLAGL

KL*

>horse

YKGEVRAPEQRKTEPVQLKTKRLREYTLKCHSSMPPNNTYVNFERFTRVV

EDVALVDQGCRALAEAHDTIQDDVEALVSIYNEKEAWYREESESARQDLS

QLRYEFRKVESLKKLLREDIQATGSSLGSMARKLDHLQVQFEALRQELSA

DLQWIQELMGSFQLESRSIDGLGSVFHRDTSESLVELFNRSCSKEVLANL

KL*

>pig

SKGESRAPEQRKAEPAQLKTKRLREYTLKCHSSMPPNNTYVNFERFARVV

EDVAQVDQGCRALSEAHDTIQDDVETLISIYNEKEAWYREESERARHDLS

QLKYEFRKVESLKKLLREDIRATGSSLGNTARKLDHLQAQFEALRQELSA

DLQWLQELVGGFQLESGSMDRLGSVFHPDTNESLVELFSRSCSKEVLAGL

QL*

>dog

SKGEVRAPEQQKTEPAQLKTKRLREYTLKCHSSMPPNNTYVNFERFARVV

EDVARVDQGCRALAGTHDTIQDDVETLISIYNEKEAWYREENESARHDLS

QLKYEFRKVESLKKVLREDIQATGSSLGSMGRKLEHLQAQFEALRQELSA

DLQWIQELVGSFQQESRNTDGLGSVFHRDARESLVELLDTSSSKEVLAGL

KL*

>bat

SKGEARAPEQRKTEPTQLKTKRLREYTLKCHSSMPPNNTYVNFERFAHVM

EDVAWVDQGCHALAGAHDTIQDDVEALVSIFNEKEAWYREESESARHDLS

QLRYEFRKVESLKRLLRKDIQATGSSLGSMARKLDHLHVQFEALRQELSA

DLQWLQELVGSCQLESGSTDGLGSVFCRDAREPLAELHSRPGSDKVLAGL

EL*

>opossum

SKGEAKALEPRKTEPPQLKTKRLREYTIKCHSSMPRNNTYVNFERFARVV

EDVADVEQSCNTLATAHDSLQDDVEALLSIYNEKEAWYREESENTRYSLS

QLKYEYRKVEAMKKALREDIQTAYSDLGSVAGKYAQLGTQYEALRRELSE

DLRWIQDLMSDFQQEKGSEESLGSDFNRDVNTSLMELLNRSCSEDFLAGL

KLRVTESSQ*

>platypus

LGGETKIPDSRAARPAQFKTKRLREYTLKCHSSMPPNNTYINFERFARAV

EDIAQAERGFSALVESRGALQADVDALVSIYNDKETWYREENENVRRDLS

RLRYEHRKVESKKKHLRDDIHGIGSRLGSVSDKYVHLGSRYESLGRELAD

HLGWIQDLMSSFQLEGRDGGCGGGNCDSVLSKDANESLMELFNRSLGQEF

LAGLKLHGADSSQ*

>chicken

LKEETKVEENKKAENTQLKTKRLREYTIKCHSSMPPNNTYINFERFARVV

EDISFMEREVSTLAASHDSLQEDIDALVSIYNEKEAWYKEENESVRHKLS

QLKYEYRKTESLKRHLQDDIKTVGASLTVITGKYAALQSQYESLSQELSE

DLKWVQDLMSNFQLENGNEACVNGNFDSVFNKDINESLTDLLNRSCCEEF

LAGLNLSVAESNQ*

>finch

LKDETKVQENKKVENTQLKTKHLREYTIKCHSSMPPNNTYINFEHFARIV

EDISRVEQGFITLAASHNSLQEDIDVLLSIYNEKEAWYKEESESVRHTLS

QLKYEYRKMESLKRHLYDDSEAVSASLAGMCGKYAELQSQYESLRQELSE

EIKWIQDLMSSFQRENETCVNGNFDSVFNKDINESLMELLNRSCCEKFLA

GLNLDATVSHQ*

>frog

SKEETKIHGTKRTAVRQLKTKRLKEYTMKSHSSMPPNNTYVNFERFAQVV

QDLSSAENEFSTLAMNYDSLQEDVEALISIYNEKETWYKEENENVRHELS

QLRYEYRKVESMKRSLHYDISSVQSGLGSLSGRYADLQSRYDSMRQELSE

DLQLIQDLVDGFHGEDAGYCGGNFASVFTRDLNESFMDLLNGSCSQDLLE

GLKIQITDSQM*

>1_stickleback

SCDHMEEESAALEAEKKAEQLKTKRLQEYTIQSHSSVPHNNTYANFERFA

HVLEDVSLMERGLSEVAVAHHSLQGDIEALLTIYNDKESWYKEESETARK

HLSQVRYEFCKVEATRRLLQEDLKSMDASLESISGKYDHRQSQLDALRQE

LSTELRWLEEVMGSLQHGRGQQQQGEYAIRNEGSAASVLQEGTEH*

>2_stickleback

PNEHKEESKAQEPKKRERRQLKTTRLREYTIKSHSSMPPNNTYVNFERFA

QVVEDIDHMEGSFASLASAHDSLQEDIDATVSIYNEKEAWYKEESEGVRH

ELSQIRYEFRKVEALKRSLQDDMRAFGSGLGAISARYRERQSHFEALRLE

LANELKWVQDVMGSFPTDGGGGGGGGYPGCSFSPVFNDDVNEALKELLNR

SRGGELLTGINLDLEPGQQR*

>1_pufferfish

SRGPVEENSAPADAEKKAEPLKTKRLKEYTIQSLSSTPQNNTYANFERFA

RVVEDVSLMEMGVSEVAEAHCALQGDMEALLSTYTGKEAWYKEESESARK

HLSQVRYEFRKVEAQRRQVQEDLQDIEAGLGSISGKFSQRQGQLDSLRLQ

LDSELQWLQEVMSSLHPEGPEGVLSGGLDTDVKQALAELLHRSCRGELYP

EARPKLTETG*

>2_pufferfish

SNEHKEETKVVDLRKRERRQLKTKRLREYTIKSHSSMPPNNTYADFERFA

QVVEDIEQMESALVSLAAAHDALQEAVDAAASACHQKEARYKEESEGVRH

ELSQIRYEFRKVEASKRSLQEDVRAFSSGLAAVAGRLRERRAHFELLRAE

LSHELAWAQEAMGSGPADGGGGCTSSAALNNDVNEALKELLSRSCEGDLL

SGIHPELDLETNKR*

>1_fugu

SRGPVEENSAPADAEKKGEPLKTKRLKEYTIQSLSSMPQNNTYANFELFA

HVVEDVSLLEVGISEVAEAHRTLQGDMEALVSIYTGKEAWYKEESESARK

HLSQVRYEFRKVEAQRRQLQEDLQDVDAGLGSISGAYSQRQRQLDSLRQE

LNSELQWLQEVMSSLHPERSGAVLGGGLNTDVKQALAELLRHSCRRELRP

EAKQKLTESG*

>2_fugu

SNEHKEENRVVDVKKRERRQLKTKRLKEYTIKSHSSMPPNNTYVNFERFA

QVVEDIEQMEGSFVSLAAAHDSLQEDVDAMASVYNEKEAWYKEESEGVRH

ELSQIRYEFRKVEALKRSLQDDVQAFSSGLAAIAGRYQERQKHLELLQAE

LSRELKWVQEVMSSCPVDGGGGGYGCAFSTALNNDVNEALTELLNRSRGG

DLLPGINLEFDIETNKR*

>1_medaka

SSGHMEEESSQCVTQKNGEKLKRTSLNEHGVLSLSSTTQNKTLFERFPLL

WEDISLIQTGLSVSADTSSFLQRDTEALLSTCNEKEAWCKETSESVQKLL

SQTCCDFQTMKATRLLLLEDVKNIDASLGSVRSDYSYRLDQLEALQQKLK

SEQLFLNPAGARSSF*

>1_medaka

SSGHMEEESSQCVTQKNGEKLKRTSLNEHGVLSLLSTTQNKTLFERFPLL

WEDISLIQTGLSVSADTSSFLQRDIEALLSTCDEKEAWCKETSESVRKLL

SQTCCDFQTMKATRLLLLEDVKNIDKSLGSVRSDYSHRLEQLEALQQKLK

SEQLFLNPAGARSSF*

>zebrafish

SNEHKEDRNKAPERKRERRQLKTKRLKEYTIKSHSSMPPNNTYINFERFA

QVEEDVSAMEGTFCQLASAHDSLQEDIDALVSIYNEKEMWYKEESESIRH

ELSQLRYEFRKVEAQRRGVHEEMRSVDASVNRVSEKYKERQSRFDALQKE

LCTELQWVQEVVGSFQVSFPNCSFSSVFNTDVNEALKELLNRSCGGDLLT

GNNLDQQR*
