# Supplementary figures and images for: New Modularity of DAP-Kinases: Alternative Splicing of the DRP-1 Gene Produces a ZIPk-Like Isoform
Source: PLoS One. 2011 Mar 8;6(2):e17344. doi: 10.1371/journal.pone.0017344 (PMC3050894; doi:10.1371/journal.pone.0017344)

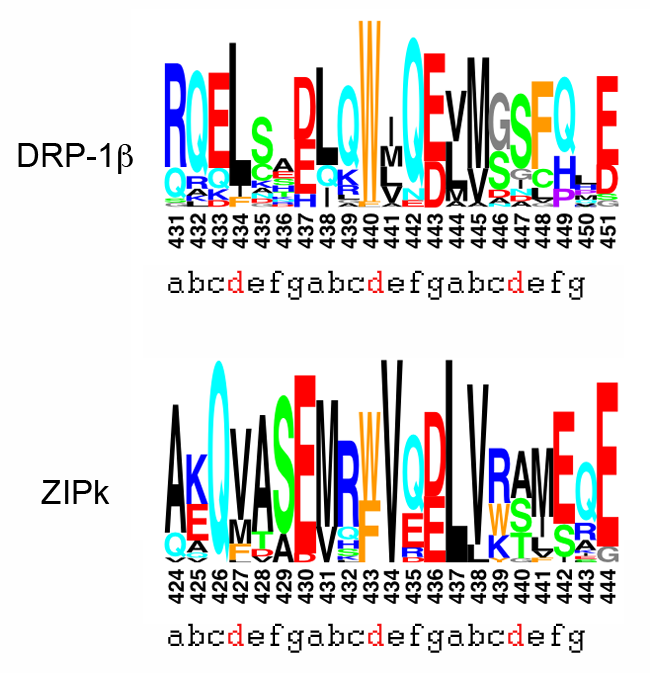

Supplement: Figure S2 — Conservation of the leucine zipper-like motif in DRP-1β and ZIPk. Logo showing the conservation of the leucine zipper-like motif of both proteins. Upper case letters colors indicate amino acid sub group; lower case letters indicate the amino acid position in the α-helix structure, with the d position of the hydrophobic amino acid marked in red. Sequence logos were calculated according to reference [Henikoff, S., Henikoff, J. G., Alford, W. J., and Pietrokovski, S. (1995) Gene (Amst.) 163, GC17–GC26]. (TIF) [file pone.0017344.s002.tif]

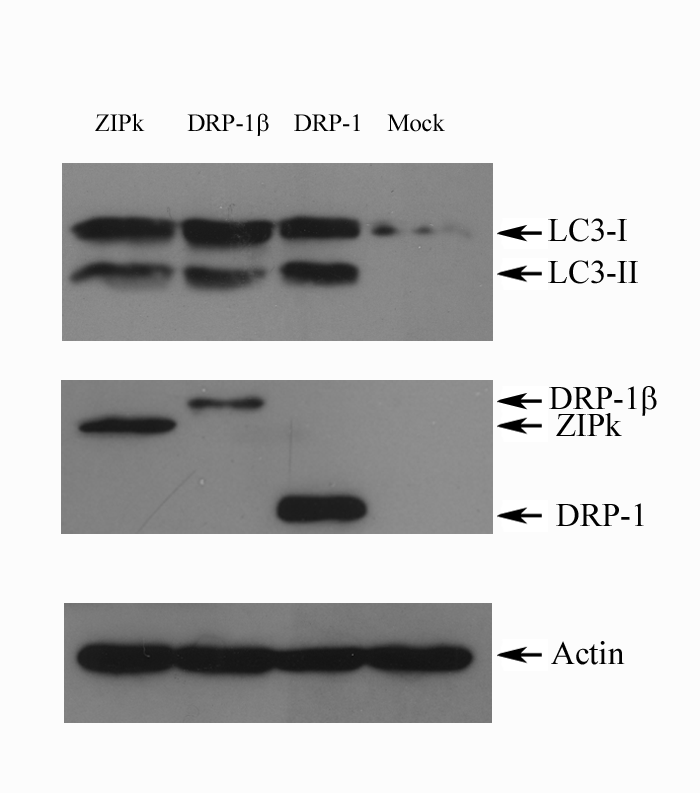

Supplement: Figure S3 — Ectopic expression of DRP-1, ZIPk and DRP-1β induces LC3 shift. HEK293T cells were transfected with the FLAG-tagged ZIPk, DRP-1β or DRP-1 expression vectors or were mock transfected with a nonrelevant protein expressing plasmind, and were harvested 24 h post transfection. Lysates were immunoblotted using anti-LC3, anti-FLAG and anti-Actin antibodies. (TIF) [file pone.0017344.s003.tif]

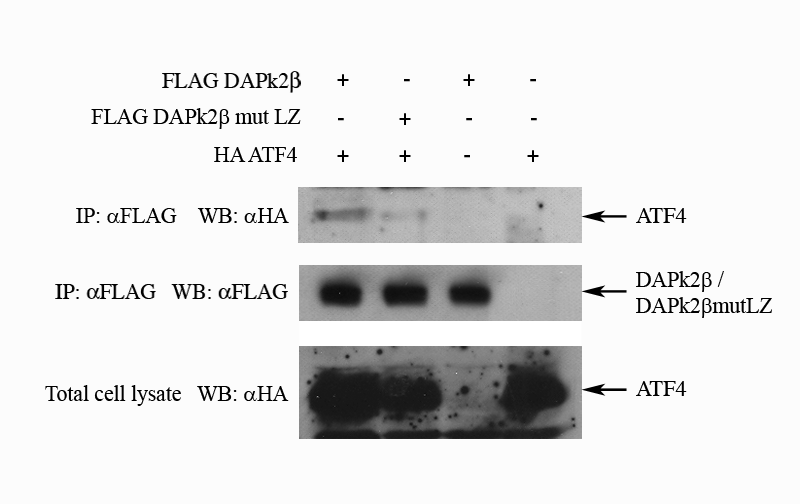

Supplement: Figure S4 — The binding of DRP-1β to ATF-4 is through the leucine zipper-like domain. HEK293T cells were co-transfected with the indicated vectors and harvested 24 h post transfection. Lysates were immunoprecipitated using anti-FLAG antibodies, and protein levels were detected using western blot with the indicated antibodies. (TIF) [file pone.0017344.s004.tif]
