# Supplementary material for: New Modularity of DAP-Kinases: Alternative Splicing of the DRP-1 Gene Produces a ZIPk-Like Isoform
Source: PLoS One. 2011 Mar 8;6(2):e17344. doi: 10.1371/journal.pone.0017344 (PMC3050894; doi:10.1371/journal.pone.0017344)
Supplement: Table S1 — Sequences used in the multiple sequence alignment of the DAP kinases. The table details organisms, sequence accession numbers and composition assembly of the sequences. (DOC) [file pone.0017344.s005.doc]

| **Organism** | | |  |
| --- | --- | --- | --- |
| **Common name** | **Short name ^a^** | **Scientific name** | **Database accession codes ^b c^** |
| **DRP-1 sequences** | | | |
| Human | Human | *Homo sapiens* | 37540936 |
| Chimpanzee | Chimp | *Pan troglodytes* | 114795056 |
| Rhesus monkey | Rhesus monkey | *Macaca mulatta* | 109158192 |
| House mouse | Mouse | *Mus musculus* | 149260095 |
| Norway rat | Rat | *Rattus norvegicus* | 62654011 |
| American pika | Pika | *Ochotona princeps* | 153575942 |
| Rabbit | Rabbit | *Oryctolagus cuniculus* | 63980343 |
| Guinea pig | Cavia | *Cavia porcellus* | 168131326 |
| Horse | Horse | *Equus caballus* | 149692810 |
| Cow ^c^ | Cow | *Bos taurus* | 119902483, 259074299 |
| Pig ^c^ | Pig | *Sus scrofa* | 194205400, 288235547 |
| Little brown bat | Bat | *Myotis lucifugus* | 105887585 |
| Dog | Dog | *Canis familiaris* | 63126605 |
| Gray short-tailed opossum | Opposum | *Monodelphis domestica* | 126278602 |
| Platypus | Platypus | *Ornithorhynchus anatinus* | 149574290, 149610442 |
| Chicken ^c^ | Chicken | *Gallus gallus* | 95059817, 25477050 |
| Zebra finch | Finch | *Taeniopygia guttata* | 212552169 |
| Western clawed frog | Silurana | *Xenopus tropicalis* | JGI_ver3.0-scaffold_62, 74310105, 133793185, 39738036, 58296577, 14184678, 71595530, 17479702, 58297420, 133793186 |
| Japanese pufferfish | Fugu | *Fugu rubripes* | 22418577 |
| Japanese pufferfish | Fugu | *Fugu rubripes* | 22418215 |
| Spotted green pufferfish | Tetraodon | *Tetraodon nigroviridis* | 47225765 |
| Spotted green pufferfish | Tetraodon | *Tetraodon nigroviridis* | 47212896 |
| Three-spined stickleback | Stickleback | *Gasterosteus aculeatus* | 86301114 |
| Three-spined stickleback ^c^ | Stickleback | *Gasterosteus aculeatus* | 86302634, 31420725, 61986721, 83712997 |
| Japanese medaka | Medaka | *Oryzias latipes strain Hd-rR* | 145797562 |
| Japanese medaka ^c^ | Medaka | *Oryzias latipes strain Hd-rR* | 187665143, 187660054, 187423073, 187524377, 145716207, 145727862, 145706148, 145709993 |
| Atlantic killifish ^c^ | Killifish | *Fundulus heteroclitus* | 152786815 |
| European seabass ^c^ | Seabass | *Dicentrarchus labrax* | 207996505 |
| European seabass ^c^ | Seabass | *Dicentrarchus labrax* | 189248886 |
| Zebrafish | Zebrafish | *Danio rerio* | 76058758 |
| Zebrafish ^c^ | Zebrafish | *Danio rerio* | 239835756, 42482726, 42430665, 126631884 |
| Spiny dogfish shark ^d^ | Dogfish shark | *Squalus acanthias* | 149774560, 110546089, 149774562, 149774561 |
| **ZIPK sequences** | | | |
| Human | Human | *Homo sapiens* | 2911155 |
| Chimpanzee | Chimp | *Pan troglodytes* | 114674686 |
| Rhesus monkey | Rhesus monkey | *Macaca mulatta* | 109122940 |
| Northern tree shrew | Tupaia | *Tupaia belangeri* | 107812812, 108226212 |
| House mouse | Mouse | *Mus musculus* | 2911153 |
| Norway rat | Rat | *Rattus norvegicus* | 3250894 |
| Ord's kangaroo rat | Kangaroo rat | *Dipodomys ordii* | 203536431, 203536432, TDB:(1527350676, 1542302641, 1556273330, 1541211393, 1578971935, 1596066105, 1562912636, 1552706462, 1569549916, 1566271599, 1585076393, 1585061768, 1542035913, 1556201828, 1541200922, 1586154871, 1585648556, 1535177412, 1569544450, 1587085697) |
| American pika | Pika | *Ochotona princeps* | 153489838, TDB(1518279754, 1536262459) |
| Guinea pig | Cavia | *Cavia porcellus* | 168075917, 168075916, 91731898, 148201962 |
| Horse | Horse | *Equus caballus* | 124068723 |
| Cow | Cow | *Bos taurus* | 119894932 |
| Little brown bat | Bat | *Myotis lucifugus* | 105860175, TDB:(976759098, 964301557, 981846460) |
| Dog | Dog | *Canis familiaris* | 73987436 |
| European shrew | Shrew | *Sorex araneus* | 80402909, 80402905, TDB:(838222606, 892417228, 873967520, 875830371, 894062723, 845045749, 848969792) |
| Cape rock hyrax | Hyrax | *Procavia capensis* | 204954140, 204954142 |
| Gray short-tailed opossum | Opposum | *Monodelphis domestica* | 84819837 |
| Platypus | Platypus | *Ornithorhynchus anatinus* | 91473547 |
| Chicken | Chicken | *Gallus gallus* | 118135662, 25936497, 25365707, TDB:227294367 |
| Zebra finch | Finch | *Taeniopygia guttata* | 197856884 |
| African clawed frog | Xenopus | *Xenopus laevis* | 66911520 |
| Western clawed frog | Silurana | *Xenopus tropicalis* | 77622189 |
| Japanese pufferfish | Fugu | *Fugu rubripes* | 22419072 |
| Spotted green pufferfish | Tetraodon | *Tetraodon nigroviridis* | 47223005 |
| Three-spined stickleback | Stickleback | *Gasterosteus aculeatus* | 86297297 |
| Japanese medaka | Medaka | *Oryzias latipes* | 145765751 |
| Fathead minnow | Fathead minnow | *Pimephales promelas* | 72422020, 73556779, 73634852, 73512480, 73721434, 72761900, 73439047, 73721433 |
| Zebrafish | Zebrafish | *Danio rerio* | 125806427 |
| Yellow perch | Perch | *Perca flavescens* | 226828992, 226825876 |
| Atlantic salmon | Salmon | *Salmo salar* | 223647734 |
| Elephant shark ^d^ | Elephant shark | *Callorhinchus milii* | 121205370, 120746673 |
| **DAPK sequences** | | | |
| Human | Human | *Homo sapiens* | 89030471 |
| Rhesus monkey | Rhesus monkey | *Macaca mulatta* | 109112050 |
| House mouse | Mouse | *Mus musculus* | 149264417 |
| Norway rat | Rat | *Rattus norvegicus* | 34873863 |
| American pika | Pika | *Ochotona princeps* | 153551468 |
| Horse | Horse | *Equus caballus* | 149755278 |
| Cow | Cow | *Bos taurus* | 119922340, 76660777 |
| Pig | Pig | *Sus scrofa* | 160952834 |
| Dog | Dog | *Canis familiaris* | 73948581 |
| Lesser hedgehog tenrec | Tenrec | *Echinops telfairi* | 72848531, 73064709, 72729470, 72672255, 72981914, 72682717, 72677720, 73198554, 72977544, 72614064, 72614065, 73320194, 73044143 |
| Gray short-tailed opossum | Opposum | *Monodelphis domestica* | 126334433 |
| Chicken | Chicken | *Gallus gallus* | 71896282, 118104157, 118104185 |
| Zebra finch | Finch | *Taeniopygia guttata* | 224088733 |
| Western clawed frog | Silurana | *Xenopus tropicalis* | JGI_ver3.0-scaffold_528 |
| Anole lizard | Anole lizard | *Anolis carolinensis* | 125788187, 125788185, 125788184 |
| Japanese pufferfish | Fugu | *Fugu rubripes* | 22418146 |
| Spotted green pufferfish | Tetraodon | *Tetraodon nigroviridis* | 47221445 |
| Three-spined stickleback | Stickleback | *Gasterosteus aculeatus* | 86292348 |
| Japanese medaka | Medaka | *Oryzias latipes strain Hd-rR* | 145743460, 145743464, 145743463, 145743461, 145743462, 17357634, 187454747, 112270082, 112347883 |
| Zebrafish | Zebrafish | *Danio rerio* | 187936992 |
| Sea lamprey ^d^ | Lamprey | *Petromyzon marinus* | (WUSTL_v.3.0:Contig19860, Contig33463)  (WUSTL_v.3.0:Contig272) |
| Florida lancelet | Amphioxus | *Branchiostoma floridae* | 227035492, 227035592, 227035493, 227035495, 227035494, 227035593, 227035594, 227035496 |
| Ciona intestinalis ^e^ | Ciona in | *Ciona intestinalis* | 26555112, 47767230, 47772066, 19475492, 24887594, 47769787, 48622814, 24872162, 24151387, 47782926, 23949821, 47959111, 47842138, 47839451, 48638142, 23937316, 24567359, 24519343, 23920607 |
| Ciona savignyi ^e^ | Ciona sa | *Ciona savignyi* | 37867999, 51735595 |
| Purple sea urchin | Sea Urchin | *Strongylocentrotus purpuratus* | 115722895 |
| Owl limpet | Limpet | *Lottia gigantea* | JGI_v1.0-sca_34 |
| Capitella | Capitella | *Capitella species strain I* | JGI_v1.0-scaffold_880 |
| C.remanei | C.remanei | *Caenorhabditis remanei* | 58824777 |
| C.briggsae | C.briggsae | *Caenorhabditis briggsae* | 22417505, 157748154, 157748152 |
| C.elegans | C.elegans | *Caenorhabditis elegans* | 13324996, 115533595, 57869091 |
| Brugia | Brugia | *Brugia malayi* | 159165084 |
| Trichinella | Trichinella | *Trichinella spiralis* | 162731928, 162731926, TDB:1724989408 |
| Honey bee | Bee | *Apis mellifera* | 110757326 |
| Jewel wasp | Wasp | *Nasonia vitripennis* | 146251437 |
| Leaf-cutting ant |  | *Atta cephalotes* | 295952817 |
| Jerdon's jumping ant |  | *Harpegnathos saltator* | 304616654 |
| Florida carpenter ant |  | *Camponotus floridanus* | 304604747 |
| Pea aphid ^f^ |  | *Acyrthosiphon pisum* | 193643384 |
| Black-legged tick ^f^ |  | *Ixodes scapularis* | 215495767 |
| *Salmon louse* ^f^ |  | *Lepeophtheirus salmonis* | 293246829, 194654331, 194658351, 290492995 |
| Starlet sea anemone ^d^ | Nematostella | *Nematostella vectensis* | TDB:(1146149752, 1146145144) |
| Fresh-water hydra ^d^ | Hydra | *Hydra magnipapillata strain 105* | (195065950, 195033723, 195063382)  (195052950) |
| **DRAK sequences** | | | |
| Human | Human | *Homo sapiens* | 3834353 |
| Human | Human | *Homo sapiens* | 3834355 |

^a^ The short names are used in the phylogenetic tree.

^b^ Accession codes are the NCBI sequences database gi (gene identifier) numbers, except for accessions from- TDB: the NCBI Trace Archive identifiers, JGI: DOE Joint Genome Institute, and WUSTL: Genome Center at Washington University in St Louis.

^c^ Accession codes of ESTs coding for the Leucine Zipper like exon of DRP-1β proteins are underlined.

^d^ The extra catalytic region for this protein is absent, and its placement with a specific gene group is due to the position of the sequence on the tree calculated from the DAPK catalytic domains (Fig. 9).

^e^ The extra catalytic region of these *Ciona* proteins is different from those of all known DAPK proteins, and its placement in with a specific gene group is due to the position of the sequences on the tree calculated from the DAPK catalytic domains (Fig. 8).

^f^ The N-terminal catalytic region and some of the extra catalytic region of these proteins is absent.
